# Recurrent chondrosarcoma of the larynx: A case report and literature review

**DOI:** 10.1097/MD.0000000000004118

**Published:** 2016-07-08

**Authors:** Hong-Wei Zhou, Jing Wang, Yang Liu, Hui-Mao Zhang

**Affiliations:** Department of Radiology, The First Hospital of Jilin University, Changchun, China.

**Keywords:** case report, conservative surgery, laryngeal chondrosarcoma, laryngeal neoplasm, laryngectomy

## Abstract

**Background:**

Laryngeal chondrosarcoma (LCS) is a rare laryngeal tumor that most commonly originates from the cricoid cartilage. The current trend for treatment of low-grade LCS is function-sparing surgical option with negative margins.

**Case summary:**

We reported here a case of a 63-year-old male patient with a 3-month history of progressive hoarseness and throat pain. The patient had undergone surgical resection of a laryngeal mass 2 years prior. A supracricoid partial laryngectomy was performed this time. Histological examination supported the diagnosis of low-grade chondrosarcoma. Three years later, the radiological and clinical findings showed no evidence of recurrence.

**Conclusion:**

Currently, total laryngectomy is preferred for patients with recurrent low-grade LCS. However, the literature review and our case suggest that a second function-preserving procedure may be a reasonable choice for recurrent LCS.

## 1 Introduction

Chondrosarcomas account for about 11% of all primary malignant bone tumors.^[1]^ Although rarely found in the larynx, they comprise about 0.1% of all neoplasms of the head and neck region whereas about 1% of all neoplasms of the larynx.^[1–3]^ The vast majority of cases (around 80%) originate from the cricoid cartilage, followed by the thyroid cartilage.^[4,5]^

The clinical manifestations of laryngeal chondrosarcoma (LCS) are nonspecific. Occasionally, patients with low-grade chondrosarcoma may be misdiagnosed with chondroma because they are closely related.^[6,7]^ Because LCS is considered a relatively low-grade tumor, a laryngeal function-preserving surgical approach is the treatment of choice in most cases.^[6]^ However, for patients with recurrent LCS, salvage laryngectomy has been recommend.^[6]^ Few studies have reported the prognosis and outcome of function-preserving surgical approach for patients with recurrent LCS.

In this report, we present the case of a 63-year-old man with recurrent low-grade LCS arising from the thyroid cartilage. This case was misdiagnosed as chondroma 2 years earlier at a local hospital. This report describes successful salvage of the neoplastic recurrence by supracricoid partial laryngectomy rather than by total laryngectomy. Three years later, the radiological and clinical findings show no evidence of recurrence.

This study was approved by the institutional review board of the First Hospital of Jilin University.

## 2 Case presentation

**Figure 1:**
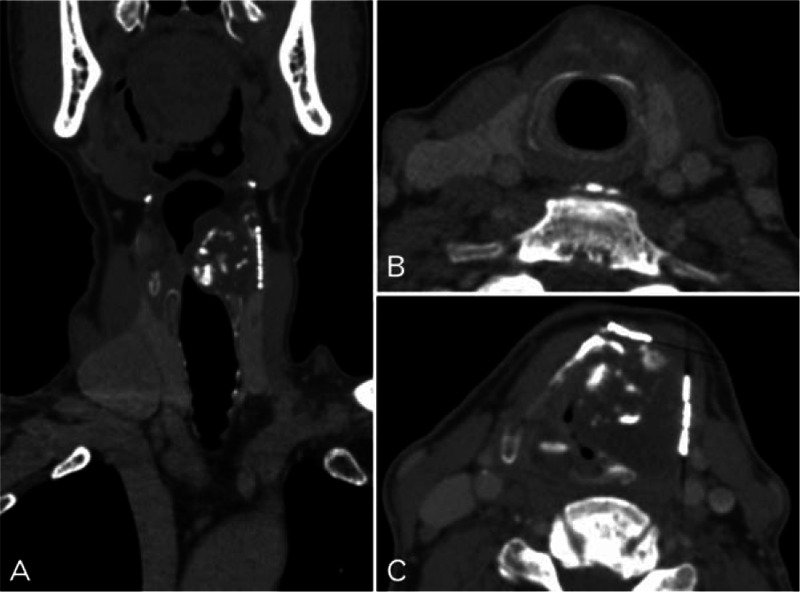
Contrast-enhanced computed tomography images of the laryngeal chondrosarcoma. Coronal (A) and axial (B and C) contrast-enhanced computed tomography images at bone windows show a mass from the thyroid cartilage, with a calcified matrix. B and C show that the cricoid cartilage and right arytenoid cartilage are free of damage.

**Figure 2:**
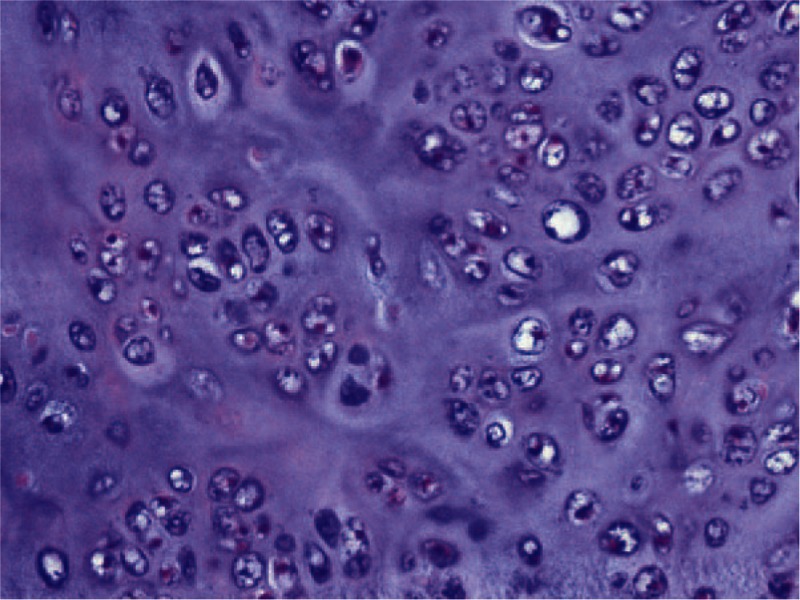
Histology of the lesion sample obtained in surgery. The image shows high-grade morphology, intermediate cellularity, and partly myxoid matrix appearance. Hematoxylin and eosin stain, ×40.

**Figure 3:**
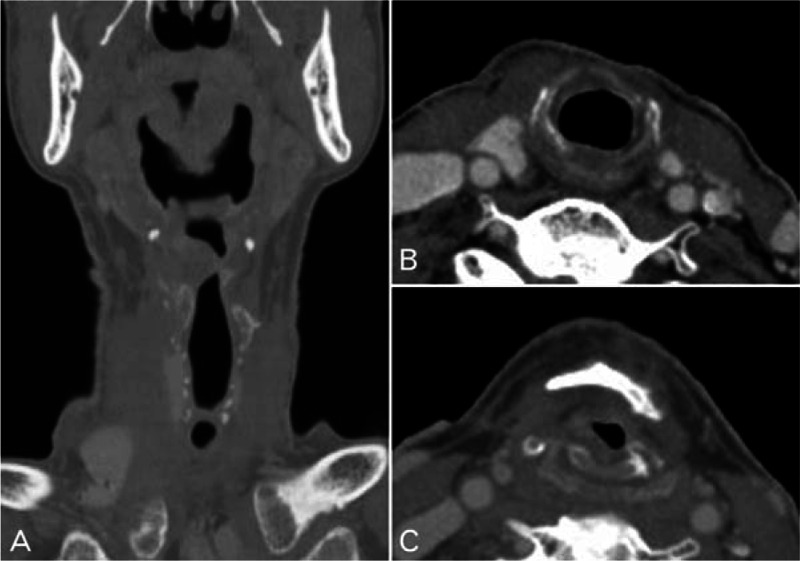
Contrast-enhanced computed tomography images 3 y after the second surgery. Images (A, B, and C) at bone windows show no signs of recurrence.

A 63-year-old man with a 3-month history of progressive hoarseness and throat pain presented to the outpatient department in May 2013. The patient had a history of surgical resection for laryngeal mass 2 years prior, which was diagnosed as laryngeal chondroma. Physical examination revealed a mass on the left side of his neck measuring approximately 3 cm in the anterior–posterior dimension. Additional lymphadenopathy was not observed in his neck. Electronic laryngoscopy revealed a spherical neoplasm with a rough surface, measuring approximately 3 cm × 3 cm. The vocal cord and the plica ventricularis were involved, while the hypolarynx was not. Computed tomography (CT) scans of the laryngeal mass showed a large, destructive tumor centered at the level of ventriculus laryngis with destruction of the thyroid cartilage and partial left arytenoid cartilage (Fig. 1). The extra-airway portion of the mass measured 3 cm × 3 cm and extended into the parapharyngeal space on the left side, compromising the airway. The intensity of the mass was heterogeneous with irregularly shaped calcification. The thyroid cartilage and parts of left arytenoid cartilage were removed during surgery. After the surgery, histological examination supported the diagnosis of LCS. Microscopic examination showed extensive distribution of cartilage formation, along with osteoclastic giant cell reaction (Fig. 2). Three years postoperatively, radiological and clinical findings revealed no evidence of recurrence (Fig. 3).

## 3 Discussion

LCS is a nonsquamous cell tumor of the larynx that accounts for only 1% of all laryngeal neoplasms. Since it was first described by Travers in 1816,^[8]^ only several hundred cases have been reported in the English literature.^[9]^

The mean age at LCS diagnosis is between 59 and 66 years with an approximately 3:1 male to female ratio.^[2,10,11]^ The etiology and histogenesis of this disease have not been clearly identified so far.^[2]^

Histological diagnosis of LCS is based on the criteria for diagnosis of malignant cartilaginous tumors described by Lichtenstein and Jaffé in 1943. These criteria were first used for malignant cartilaginous tumors of extralaryngeal bone origin.^[12]^ In 1977, Evans et al grouped chondrosarcomas into grades I, II, and III according to the mitotic rate, cellularity, and nuclear size. Higher histological grades are associated with poor prognosis.^[13]^ This is the most widely used classification criteria for LCS.^[6]^ Vimentin and S-100 protein expression may be helpful for diagnosis of high-grade LCS.^[14]^

High-resolution CT with bone windows and contrast is an essential and complementary investigative technique for accurately delineating the extent of the tumor preoperatively. It is useful for optimizing tumor management.^[15]^ On high-resolution CT, LCS of the current case had a destructive lesion with an enlarged soft mass and irregular erosion of the thyroid cartilage and partial left arytenoid cartilage. Furthermore, high-resolution CT images may reveal varying degrees of intratumoral calcifications. In our case, the patient had intratumoral calcifications, with the erosion of the ventriculus laryngis. Compared to CT, magnetic resonance imaging may more clearly show the extent of the tumor.^[6]^

Low-grade LCSs are most common, while moderate- and high-grade tumors are less common. Low-grade LCS grows slowly, rarely metastasize, and may have a clinical course similar to chondromas.^[6]^ However, chondrosarcomas tend to recur without radical surgical procedures. Many of these tumors could be overlooked and may grow to considerable size before correct diagnosis is made.^[16,17]^

Considering the natural history, conservative surgery has been the preferred surgical option for LCS in recent years.^[9,18,19]^ Rather than total laryngectomy, the surgical methods such as CO_2_ laser resection, hemicricoidectomy, or hemilaryngectomy are the treatment of choice for low-grade LCS. Conservative surgery can preserve the structural and functional integrity of the larynx, which is very important for patient quality of life. However, total laryngectomy is the preferred treatment for high-grade LCS.^[2,3]^

Literature reviews suggest an LCS recurrence rate of 16% to 18%.^[10,20]^ For patients with recurrent LCS, total laryngectomy is also recommend.^[2,19]^ There has been some evidence that the efficacy of total laryngectomy for recurrent LCS is comparable to that of initial total laryngectomy.^[21]^ However, there is little evidence on the efficacy of conservative surgery for recurrent LCS. Sauter et al reported a case of a 93-year-old male patient with recurrent low-grade LCS, in which a second function-preserving surgery was performed 1 month after the initial surgery. Follow-up 3 months after the second surgery showed no evidence of recurrence. Pelliccia et al reported that repeated endoscopic resection was effective for patients with recurrent cricoid chondrosarcoma (1 patient).^[22]^ Other case reports also support the use of second function-preserving procedures for patients with recurrent LCS.^[23,24]^ Our case developed recurrence 2 years after the initial conservative surgery. Although total laryngectomy was recommended by the surgeons, a second conservative surgery was performed because the patient insisted that quality of life was more important. Three years after the second surgery, there was no evidence of recurrence.

## 4 Conclusion

LCS is a rare laryngeal tumor that most commonly originates from the cricoid cartilage. Function-sparing surgery with negative margins is the current treatment of choice for low-grade LCS. It allows the radical removal of the tumor through a larynx-preserving procedure, thus representing a valid alternative to total laryngectomy. Currently, for patients with recurrence of low-grade LCS, total laryngectomy is preferred. However, a second function-preserving procedure may be also a reasonable choice, especially for older patients or those with serious comorbidities. However, total laryngectomy remains the preferred treatment for high-grade LCS.
